# Comparative Study of Essential Oils Extracted from Egyptian Basil Leaves (*Ocimum basilicum* L.) Using Hydro-Distillation and Solvent-Free Microwave Extraction

**DOI:** 10.3390/molecules21010113

**Published:** 2016-01-19

**Authors:** Mohammed Chenni, Douniazad El Abed, Njara Rakotomanomana, Xavier Fernandez, Farid Chemat

**Affiliations:** 1 Laboratoire de Chimie Fine, Département de Chimie, Faculté des Sciences Exactes et Appliquées, Université d’Oran 1 Ahmed Ben Bella, B.P. 1524, El M’Naouer, Oran 31000, Algeria; chenni.mohamed@hotmail.fr; 2 GREEN Extraction Team, INRA, UMR 408, Université d’Avignon et des Pays du Vaucluse, Avignon 84000, France; njara.rakotomanomana@univ-avignon.fr (N.R.); farid.chemat@univ-avignon.fr (F.C.); 3 Institut de Chimie de Nice, UMR 7272, Université de Nice-Sophia Antipolis/CNRS, Parc Valrose, Nice 06108, France; xavier.fernandez@unice.fr

**Keywords:** basil (*Ocimum basilicum* L.), microwave, hydro-distillation, essential oil, chemical composition, GC/MS, antioxidant activity, DDPH assay, antimicrobial activity

## Abstract

Solvent-free microwave extraction (SFME) and conventional hydro-distillation (HD) were used for the extraction of essential oils (EOs) from Egyptian sweet basil (*Ocimum basilicum* L.) leaves. The two resulting EOs were compared with regards to their chemical composition, antioxidant, and antimicrobial activities. The EO analyzed by GC and GC-MS, presented 65 compounds constituting 99.3% and 99.0% of the total oils obtained by SFME and HD, respectively. The main components of both oils were linalool (43.5% SFME; 48.4% HD), followed by methyl chavicol (13.3% SFME; 14.3% HD) and 1,8-cineole (6.8% SFME; 7.3% HD). Their antioxidant activity were studied with the 2,2-diphenyl-1-picrylhydrazyl (DPPH^•^) radical scavenging method. The heating conditions effect was evaluated by the determination of the Total Polar Materials (TPM) content. The antimicrobial activity was investigated against five microorganisms: two Gram-positive bacteria, *Staphylococcus aureus* and *Bacillus subtilis*, two Gram-negative bacteria, *Escherichia coli* and *Pseudomonas aeruginosa*, and one yeast, *Candida albicans*. Both EOs showed high antimicrobial, but weak antioxidant, activities. The results indicated that the SFME method may be a better alternative for the extraction of EO from *O. basilicum* since it could be considered as providing a richer source of natural antioxidants, as well as strong antimicrobial agents for food preservation.

## 1. Introduction

Aromatic plants and EOs have been used since ancient times and are still widely used for their biological properties [1,2] and their applications in various industries: food, cosmetics, perfumery, and pharmacy [3]. Nowadays, EOs are attracting substantial interest from scientists because of their use in the treatment of certain infectious diseases for which synthetic antibiotics are becoming less and less active, or for preserving food against oxidation as alternatives to synthetic chemicals.

*Ocimum basilicum* L., named commonly as sweet basil, is a popular culinary herb belonging to the Lamiaceae family. *O. basilicum* known as *lahbeq* in Algeria [4] and called *rehan* in Arabic [5], is originally native to India and other Asian regions. Today, it is cultivated all over the world [6]. Traditionally, the basil leaves are used in folk medicine as a remedy for a large number of diseases, including cancer, convulsion, diarrhea, epilepsy, gout, nausea, sore throat, toothaches, and bronchitis [7,8,9]. It is also a source of EO containing biologically-active constituents which possess antioxidant and antimicrobial properties [3,5,6,7,8,9]. The chemical composition of basil oil has been the subject of several studies. Basil oils have been classified into four chemotypes according to their chemical composition and geographical source. The European type, cultivated in Europe, USA, and Africa, is characterized by linalool and methyl chavicol as the major oil constituents. The Reunion type, located in the Comoros and Seychelles Islands, Africa, and Reunion Island, is characterized by a high concentration of methyl chavicol. Tropical type originated from India, Pakistan, Guatemala, Haiti, and Africa is rich in methyl cinnamate. Another basil chemotype, with eugenol as the main component, is common in North Africa, Russia, Eastern Europe, and parts of Asia [6,10,11,12]. In addition to these, other basil oils have also been reported which contained various quantities of linalool, camphor, methyl chavicol, methyl cinnamate, and eugenol [13]. EOs are obtained from plants by various conventional and unconventional processes, such as mechanical pressing, maceration, solvent extraction, supercritical fluid and subcritical water extractions, and HD [14,15]. An original method, the solvent-free microwave extraction (SFME), has been developed and was patented in 2004 [16,17]. SFME is one of the newest techniques for EOs extraction assisted by microwaves, without any solvent or water, at atmospheric pressure. The advantages of using microwave energy are correlated to its effective heating and faster energy transfer and the fact that it is environmentally friendly [18,19,20]. To the best of our knowledge, there are no previous reports about the comparison of the chemical composition, antioxidant, and antimicrobial activities of the EO of *O. basilicum* L. leaves isolated by SFME and HD. Therefore, the aim of the present study is to provide data from the comparison between SFME and conventional HD in terms of extraction time, yield, chemical composition, antioxidant, and antimicrobial activities of EO extracted from Egyptian basil leaves.

## 2. Results and Discussion

### 2.1. Kinetics of Essential Oils Extracted by SFME and HD

**Figure 1:**
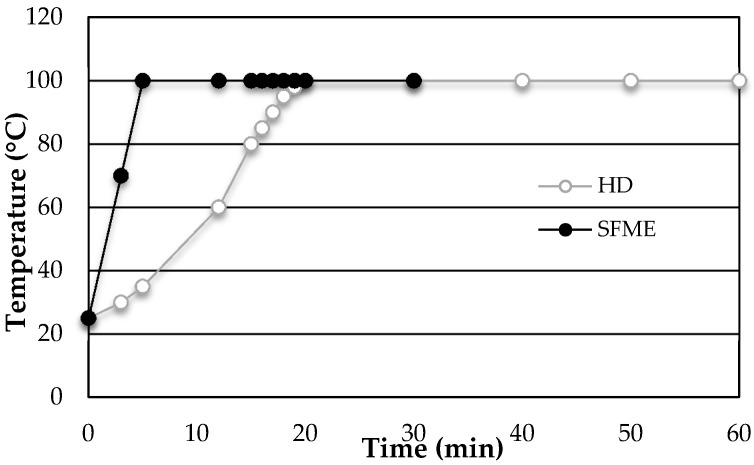
Temperature profiles as a function of time for the SFME (●) and HD (○) extraction of EO from sweet basil leaves.

**Figure 2:**
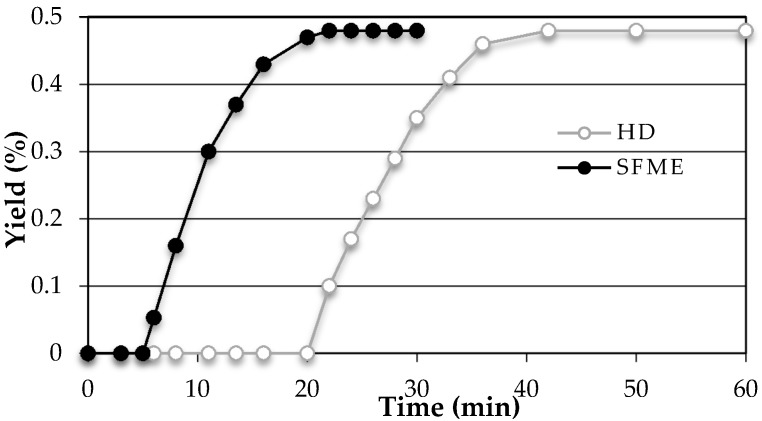
Yield profiles as a function of time for the SFME (●) and HD (○) extraction of EO from sweet basil leaves.

Figure 1 and Figure 2 represent the kinetics of EO obtained by SFME and HD from dry basil leaves. During the extraction process, the system temperature is equal to the boiling temperature of water at atmospheric pressure (100 °C). To reach this temperature, and thus obtain the distillation of the first EO droplet, it is necessary to heat the sample for only 5 min with SFME against 20 min for HD (Figure 1).

As shown on Figure 2, an extraction time of 30 min with SFME provides similar yields (0.48% ± 0.02%) to those obtained after 60 min by HD. The results show that SFME is clearly advantageous with respect to conventional HD in terms of rapidity, efficiency, and substantial energy savings.

### 2.2. Evaluation of Physical and Organoleptic Properties

Physical properties (specific gravity, refractive index, and solubility) and sensory evaluation of EO extracted by SFME and HD from the basil samples are shown in Table 1. There are no significant differences observed between the physical constants of the EO obtained by these two methods. The only difference resides in the color of the EO extracted by SFME which was lighter than that isolated by the classical method. The SFME method offers the possibility for a better reproduction of natural aroma of basil essential oil than the HD method.

**Table 1:** Physical and organoleptic properties of sweet basil EO obtained by SFME and HD.

	Extraction Methods	
	SFME-EO	HD-EO
**Physical Properties**		
Specific gravity (20 °C)	0.926	0.917
Refractive index (20 °C)	1.486	1.480
Solubility	Water insoluble. Soluble in alcohol and other organic liquids	
**Organoleptic Properties**		
Color	Pale yellow	Yellow-greenish
Odor	Sweet minty pleasant odor	Strong and pungent minty odor
Aspect	Mobile liquid	Mobile liquid

### 2.3. Chemical Composition of the Essential Oils Obtained by SFME and HD

The components identified by GC and GC-MS in basil (EOs) from both methods are reported in Table 2. The EO of basil leaves isolated either by SFME or HD contains the same dominant components. Among 65 compounds identified in basil EO, representing 99.3 and 99.0% of the total oils obtained by SFME and HD, respectively, monoterpene, sesquiterpene, and derivatives of phenylpropanoid compounds were identified. Monoterpenes are the main components in the EOs but the relative amounts differ for the two extraction methods. The oxygenated compounds: monoterpene alcohol linalool is the most abundant component in basil EO (43.5% for SFME and 48.4% for HD) followed by methyl chavicol (13.3% for SFME and 14.3% for HD) and 1,8-cineole (6.8% for SFME and 7.3% for HD). Slightly lower amounts of oxygenated compounds (89.8% *vs.* 89.6%) and higher amounts of hydrocarbons (9.7% *vs.* 9.2%) are present in the EO of basil extracted by SFME in comparison with HD. The chemical profile of the basil EO, isolated by SFME and HD in our study, was comparable to that reported by Figueredo *et al.* [21].

**Table 2:** Chemical composition of the Egyptian basil essential oils leaves obtained by SFME and HD.

No	Compounds ^1^	LRI_HP1_^2^	LRI_INNO_^2^	HD-EO (% ± SD)	SFME-EO (% ± SD)	Identification Methods
**Monoterpenes Hydrocarbons**				1.9	1.3	
1	Tricyclene	915	1017	tr	tr	LRI, MS
2	α-Thujene	919	1030	0.1	0.1	LRI, MS
3	α-Pinene	929	1070	0.2	0.1	LRI, MS, Std
4	Camphene	940	1072	0.1	0.1	LRI, MS, Std
5	Sabinene	962	1125	0.2	0.2	LRI, MS, Std
6	β-Pinene	969	1114	0.4	0.3	LRI, MS, Std
7	β-Phellandrene	981	1167	0.3	0.2	LRI, MS, Std
8	(*E*)-β-Ocimene	1034	1249	0.2	0.1	LRI, MS, Std
9	γ-Terpinene	1048	1245	0.4 ± 0.1	0.2	LRI, MS, Std
10	Terpinolene	1075	1285	tr	tr	LRI, MS, Std
**Oxygenated Monoterpenes**				83.2	82.9	
11	1,8-cineole	1026	1212	7.3 ± 0.3	6.8 ± 0.2	LRI, MS, Std
12	Linalool	1095	1552	48.4 ± 0.9	43.5 ± 0.8	LRI, MS, Std
13	Camphor	1130	1523	0.3	0.4 ± 0.1	LRI, MS, Std
14	Menthone	1145	1480	0.3 ± 0.1	0.1	LRI, MS, Std
15	Borneol	1160	1700	0.8 ± 0.1	1.0 ± 0.1	LRI, MS, Std
16	Methyl chavicol	1181	1167	14.3 ± 0.4	13.3 ± 0.2	LRI, MS, Std
17	Fenchyl acetate	1198	1480	0.2	0.1	LRI, MS
18	Citronellol	1210	1760	tr	tr	LRI, MS, Std
19	Cuminaldehyde	1213	1780	tr	tr	LRI, MS
20	Neral	1215	1670	tr	tr	LRI, MS
21	Carvone	1217	1732	0.1	tr	LRI, MS, Std
22	Chavicol	1231	2325	0.1	tr	LRI, MS
23	Geraniol	1235	1841	0.2	0.1	LRI, MS, Std
24	Linalyl acetate	1241	1556	0.1	tr	LRI, MS, Std
25	Anethol	1264	1825	0.7	0.6	LRI, MS, Std
26	Bornyl acetate	1271	1590	1.5 ± 0.1	1.1	LRI, MS, Std
27	(*Z*)-Methyl cinnamate	1274	1969	0.5 ± 0.1	0.4 ± 0.1	LRI, MS, Std
28	Myrtenyle acetate	1299	1688	tr	tr	LRI, MS
29	Eugenol	1330	2155	2.4 ± 0.1	2.9 ± 0.1	LRI, MS
30	(*E*)-Methylcinnamate	1356	2091	2.3 ± 0.1	6.5 ± 0.1	LRI, MS, Std
31	Methyl eugenol	1371	1989	3.7 ± 0.1	6.1 ± 0.1	LRI, MS, Std
**Sesquiterpenes Hydrocarbons**				7.3	8.4	
32	α-Cubebene	1342	1463	tr	tr	LRI, MS
33	β-Bourbonene	1378	1542	0.2	0.2 ± 0.1	LRI, MS
34	β-Elemene	1380	1589	0.7 ± 0.1	0.9 ± 0.1	LRI, MS
35	α-Copaene	1381	1490	0.1	0.2	LRI, MS, Std
36	β-Caryophyllene	1411	1602	0.1	0.1	LRI, MS, Std
37	α-Cedrene	1412	1589	tr	tr	LRI, MS
38	β-Cubebene	1413	1545	0.1	tr	LRI, MS
39	α-Bergamotene	1430	1568	2.5 ± 0.1	2.7 ± 0.2	LRI, MS
40	α-Humulene	1445	1667	0.2 ± 0.1	0.3 ± 0.1	LRI, MS, Std
41	α-Guaiene	1447	1597	tr	tr	LRI, MS
42	β-Farnesene	1457	1698	0.4	0.4 ± 0.1	LRI, MS
43	α-Curcumene	1470	1782	tr	tr	LRI, MS
44	γ-Muurolene	1473	1669	0.1	0.2	LRI, MS
45	Alloaromadendrene	1475	1637	0.3	0.4 ± 0.1	LRI, MS
46	Germacrene D	1478	1705	0.8	0.9 ± 0.1	LRI, MS
47	δ-Guaiene	1492	1715	0.2 ± 0.1	0.1	LRI, MS
48	γ-Cadinene	1505	1757	1.1 ± 0.1	1.3 ± 0.1	LRI, MS
49	Calamenene	1508	1830	0.5	0.7 ± 0.1	LRI, MS
**Oxygenated Sesquiterpenes**				6.5	6.6	
50	β-Ionone	1455	1920	0.1	tr	LRI, MS, Std
51	Nerolidol	1538	2009	0.3 ± 0.1	0.4 ± 0.2	LRI, MS, Std
52	Spathulenol	1561	2131	0.4 ± 0.1	0.6 ± 0.1	LRI, MS
53	Caryophyllene oxide	1565	1981	0.2	0.3	LRI, MS
54	Carotol	1592	2006	0.5	0.8 ± 0.1	LRI, MS
55	Cadinol ^3^	1615	2147	0.8 ± 0.1	0.9 ± 0.2	LRI, MS
56	τ-Cadinol	1629	2165	0.1	0.1	LRI, MS
57	α-Cadinol	1637	2201	tr	tr	LRI, MS
58	α-Bisabolol	1650	2215	4.1 ± 0.1	3.5 ± 0.1	LRI, MS
59	Phytol	2080	-	tr	tr	LRI, MS
**Other Oxygenated Compounds**				0.1	0.1	
60	Methyl 2-methylbutyrate	758	1008	tr	-	LRI, MS
61	Hexanal	773	1090	tr	tr	LRI, MS, Std
62	(*Z*)-2-Hexenal	823	1120	tr	tr	LRI, MS, Std
63	Benzyl benzoate	1710	2571	tr	tr	LRI, MS, Std
64	6,10,14-Trimethyl pentadecan-2-one	1816	-	0.1	0.1	LRI, MS
65	Farnesyl acetone	1867	2382	tr	tr	LRI, MS
Extraction time				60 min	30 min	
Yields %				0.48% ± 0.02%	0.48% ± 0.02%	
Total oxygenated compound				89.8	89.6	
Total non-oxygenated compound				9.2	9.7	
Total identified compound				99.0	99.3	

^1^ Compounds are listed in order of their classes. Compositional values less than 0.1% are denoted as traces (tr). Presence of a compound is indicated by its GC-FID percentage with S.D. = standard deviation, absence is indicated by “-”; ^2^ RI = retention indices are determined on HP-1 and INNOWAX column using the homologous series of n-alkanes (C8–C24); ^3^ Correct isomer not identified.

A substantial number of studies conducted on the composition of the EO of basil revealed a huge diversity in the constituents of its oil with different chemotypes from many regions of the world. It can be described from Table 3 that the major constituents which have been isolated from different *O. basilicum* oils include linalool, methyl chavicol, eugenol, methyl cinnamate, 1,8-cineole, bergamotene, limonene, camphor, α-cardinol, geraniol, *etc.* [22]. In the Brazilian basil leaf EOs [23], linalool, geraniol, and 1,8-cineole are the major compounds corroborating with Oman basil [5], as well as Poland [24] basil. For Czech Republic [25], Guinea [26], and Reunion [18], the major compounds of basil EO are linalool and eugenol. Marotti *et al.* [10] reported the presence of linalool, methyl chavicol, and eugenol as main components of Italian basil EO. It was also reported that four major compounds characterize the basil EO of Austria: linalool, methyl chavicol, methyl cinnamate, and α-cadinol [27]. These results are consistent with those obtained in previous works in Bulgaria [28] and USA [29]. In *O. basilicum* EO from Romania [30], linalool was reported as the main component. The major compounds of EO extracted from Algeria [31] leaves of basil, were linalool, linalyl acetate, elemol, and geranyl acetate. However, the first major compound from French Polynesia basil leaf EOs [32] is methyl cinnamate. Regarding Pakistan basil [33], four major compounds were found: linalool, epi-α-cardinol, α-bergamotene, and γ-cadinene. In the case of Egypt basil oils, Ismail [34] has reported linalool, 1,8-cineole, eugenol, and methyl cinnamate as dominant components. Concerning basil from Turkey, Chalchat *et al.* [35] cited three major compounds: methyl chavicol, limonene, and *p*-cymene. Additionally, the EO from Madagascar [36], Iran [37], and Thailand [38] is rich in methyl chavicol. On the other hand, Purkayastha [39] reported that camphor, followed by limonene and β-selinene were the major compounds in *O. basilicum* EO from Northeast India. According to Simon *et al.* [6], the Egyptian basil is very similar to the European, characterized by linalool and methyl chavicol as the major oil constituents that were in agreement to our results that the essential oil of basil belonged to the linalool-rich type. The observed difference in the constituents of basil EOs between countries may be probably due to environmental conditions and genetic factors, different chemotypes, and the nutritional elements of the plants, as well as other factors that can influence the oil composition.

**Table 3:** Composition of the main compounds of *O. basilicum* L. essential oil from different countries.

Country	Part Used	Major Constituents (%)	Reference
Oman	Plants	Linalool (69.9), geraniol (10.9), 1,8-cineole (6.4), α-bergamotene (1.6), geranyl acetate (1.4)	[5]
Italy	Fresh aerial parts	Linalool (41.17–76.20), methyl chavicol (18.01–41.40), eugenol (1.16–3.89), 1,8-cineole (0.94–12.91)	[10]
Reunion	Fresh plants	Linalool (25.3–39.1), eugenol (11.0–43.2), *trans*-α-bergamotene (6.0–7.6)	[18]
Brazil	Dried leaves	Linalool (72.14), geraniol (12,95), 1,8-cineole (7.90)	[23]
Poland	Dried plants	Linalool (64.7), geraniol (12.6), 1,8-cineole (4.1), epi-α-cadinol (3.8)	[24]
Czech Republic	Fresh leaves	Linalool (15.6–32.2), eugenol (9.1–22.2), 1,8-cineole (3.1–20.2), bergamotene (1–20.2)	[25]
Guinea	Plants	Linalool (69.0), eugenol (10.0), (E)-α-bergamotene (3.0), thymol (2.0)	[26]
Austria	Dried leaves	Linalool (28.6), methyl chavicol (21.7), (*E*)-methyl cinnamate (14.3), α-cadinol (7.1), eugenol (5.9), 1,8-cineole (4.0)	[27]
Bulgaria	Dried leaves	Linalool (54.95), methyl chavicol (11.98), methyl cinnamate (7.24)	[28]
USA	Dried leaves	Linalool (3.94), methyl chavicol (2.03), methyl cinnamate (1.28)	[29]
Romania	Dried plants	Linalool (46.95), β-elemene (7.84), farnesene (6.86), epi-bicyclosesquiphellandrene (5.92), α-guaiene (5.26)	[30]
Algeria	Dried leaves	Linalool (32.83), linalyl acetate (16), elemol (7.44), geranyl acetate (6.18), myrcene (6.12), allo-ocimene (5.02), α-terpineol (4.9), (*E*)-β-ocimene (3.68), neryl acetate (3.45)	[31]
French Polynesia	Fresh leaves	(*E*)-methyl cinnamate (43.4–62.3), (*Z*)-methyl cinnamate (8.1–8.6), linalool (4.6–21.9)	[32]
Pakistan	Dried aerial parts	Linalool (56.7–60.6), epi-α-cadinol (8.6–11.4), α-bergamotene (7.4–9.2),γ-cadinene (3.2–5.4)	[33]
Egypt	Aerial parts	Linalool (44.18), 1,8-cineole (13.65), eugenol (8.59), methyl cinnamate (4.26), iso-caryophyllene (3.10), α-cubebene (4.97)	[34]
Turkey	Flower, leaves, stem	Methyl chavicol (58.26, 52.60, 15.91), limonene (19.41, 13.64, 2.40), *p-*cymene (0.38, 2.32, 2.40)	[35]
Madagascar	Plants	Methyl chavicol (74–87), 1,8-cineole (2.55–4.43), linalool (0.97–2.72), methyl eugenol (0.87–4.16)	[36]
Iran	Dried leaves	Methyl chavicol (46.9), geranial (19.1), neral (15.15), geraniol (3.0), nerol (3.0), caryophyllene (2.4)	[37]
Thailand	Aerial parts	Methyl chavicol (92.48), β-ocimene (2.27), *trans*-α-bergamotene (2.14)	[38]
India	Aerial parts	Camphor (42.1), limonene (7.6), β-selinene (5.6)	[39]

### 2.4. Antioxidant Activity

**Figure 3:**
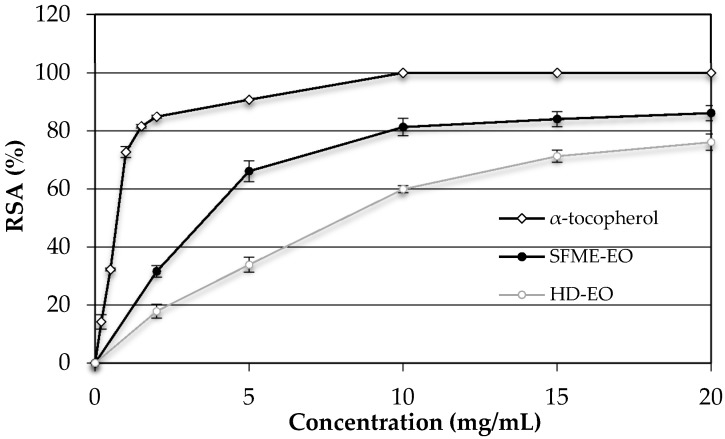
Evaluation of antioxidant activity of basil essential oils (● SFME-EO ○ HD-EO ◊ α-tocopherol).

The free radical scavenging activity of basil EOs was measured by the DPPH assay. The DPPH**˙** radical is one of the most commonly used substrates for the fast evaluation of antioxidant activity because of its stability in radical form and the simplicity of the assay. *O. basilicum* EOs were able to reduce the stable, purple-colored radical DPPH**˙** into yellow-colored DPPH-H [40]. The results of antioxidant activity of *O. basilicum* EO are reported in Figure 3 (the experiments were done in triplicate). It can be seen that *O. basilicum* EO extracted by SFME exhibited a dose dependent increase with a radical scavenging effect of 86.13% ± 2.8% at 20 mg/mL, which is higher than the DPPH% inhibition of the *O. basilicum* EO extracted by HD (76.13% ± 2.6%) at the same concentration. The DPPH% inhibition of the α-tocopherol (90.74% ± 0.2%) at 5 mg/mL is higher than the DPPH% inhibition of the *O. basilicum*. DPPH scavenging activity is usually presented by the value of IC_50_ (concentration that inhibits 50% of the DPPH radical). A comparison between the DPPH scavenging activity of *O. basilicum* EOs (SFME-EO IC_50_ = 3.6 mg/mL, HD-EO IC_50_ = 8.1 mg/mL) and those expressed by α-tocopherol (IC_50_ = 0.8 mg/mL) showed that the EO exhibited weakest antioxidant effects than α-tocopherol (vitamin E). Therefore, the antioxidant effect of the oil was about four times lower for SFME-EO and 10 times lower for HD-EO than that of the standard antioxidant. Previous studies have shown that *O. basilicum* has potent antioxidant properties [5,41,42,43,44,45]. Our findings are in agreement with Hadj-Khelifa *et al.* [31] who reported that *O. basilicum* EO containing linalool as a major compound exhibited lower antioxidant effects (IC_50_ = 83.4 mg/mL) than the vitamin E (IC_50_ = 22.0 mg/mL). Hussain *et al.* [33] showed that sweet basil EO which is rich in linalool offered antioxidant activity comparable to synthetic antioxidant BHT. However, Bozin *et al.* [46] found that basil oil containing methyl chavicol (45.8%) and linalool (24.2%) as major components exhibited very strong RSA, reducing the DPPH radical formation (IC_50_) in the range of 0.4 μg/mL, and indicated that the most responsible compounds were the oxygenated phenolic monoterpene methyl chavicol and the mixture of mono and sesquiterpene hydrocarbons. Quite a different situation was observed by Mahmoud *et al.* [47], which found that the main component methyl chavicol exhibit a moderate antioxidant activity (DPPH assay). Also, Dawidowicz *et al.* [48] which showed that the main component methyl chavicol, do not exhibit antioxidant properties. The antioxidant properties of basil EOs (I% about 46.1% ± 1.3%) result from the properties of other components, especially methyl eugenol that was considered as the main contributors of the antioxidant activity in the EO of *Myrtus communis* L. [49]. Politeo *et al.* [27] clearly suggests that the antioxidant capacity (IC_50_ values of 1.4 g/L) of total EO is due only or mainly to the presence of eugenol (5.9%) in its chemical composition and that other constituents do not have significant effect on eugenol capacity. The antioxidant activity of eugenol has been reported several times, tested on various systems [50,51,52]. In accordance, Pripdeevech *et al.* [53] reported that the EO of *O. basilicum* from Thailand (linalool/eugenol chemotype) exhibited high scavenging ability of DPPH radicals (IC_50_ = 26.53 ± 0.94 μg/mL). The same finding was reported by Dabire *et al.* [54] who showed that the decrease in the rate of eugenol in EO of *O. basilicum* causes a decrease of its antioxidant power. Lee *et al.* [29] also found that eugenol (0.896 mg/g) was the main contributor of the antioxidant activity of volatile extract of basil than major components. Hence, the main component does not always determine the antioxidant activity of the examined EOs. It is possible that components present at lower concentrations might be involved in some type of synergy with other active compounds. Our study indicated than the antioxidant activity of SFME-EO was relatively more active than HD-EO. As a result, the antioxidant activity of *O. basilicum* EO may be attributed to the higher content of linalool, methyleugenol, eugenol, and other minor components.

### 2.5. Oil Enrichment and Heating Test

**Figure 4:**
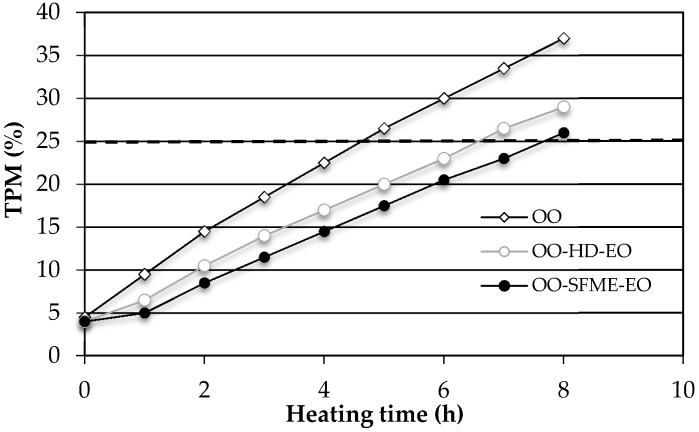
Comparison of TPM during the frying process (at 180 °C) of olive oil containing basil essential oils (● OO-SFME-EO; ○ OO-HD-EO) and virgin olive oil (◊ OO).

All the samples containing OO displayed close TPM values before heating but the total heating times required to reach the maximal TPM value of 25% were quite different: 4.7 ± 0.1 h for OO, 6.6 ± 0.1 h for OO-HD-EO and 7.8 ± 0.1 h for OO-SFME-EO (Figure 4). Hence, the efficient EO supplementation of OO would significantly prolong the OO shelf life. The results show a high level of stability of virgin OO enriched with basil EO, especially those isolated by SFME. These results are in good agreement with other findings in the literature [55,56,57], which TPM effect might be attributed to the phenolic compounds present in the EO, although there is no specific report carried out about basil oils on fried OO.

### 2.6. Antimicrobial Activity

**Figure 5:**
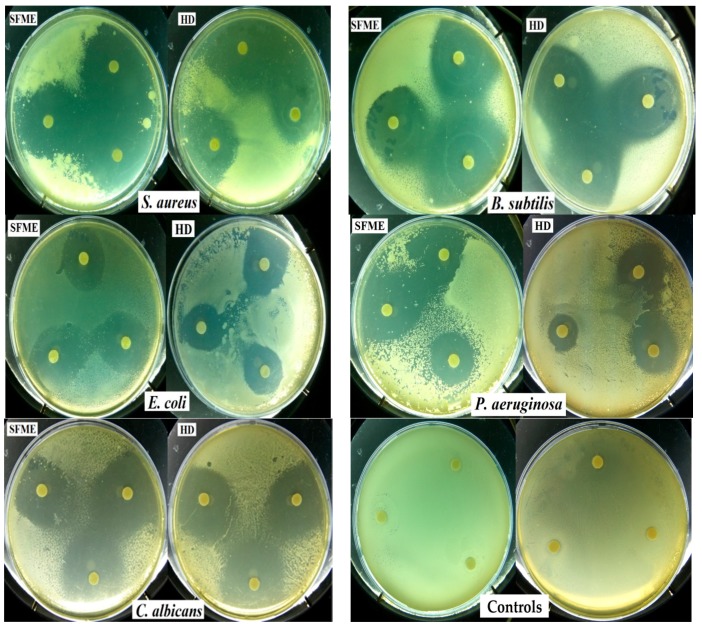
Photograph showing inhibition zones of the microbial growth of all tested microorganisms and control.

The antimicrobial activity of sweet basil oil was tested against five pathogenic microorganisms. The results obtained from disc diffusion method (Figure 5), followed by measurement of minimum inhibitory concentration (MIC) represented in Table 4, indicated that *S. aureus* was the most sensitive microorganisms showing largest inhibition zones (38–33 mm) and lowest MIC values (18–25 µL/mL). The lowest activity was observed against *E. coli* with the smallest inhibition zones (26–22 mm) and highest MIC value (25–30 µL/mL). Furthermore, growth inhibition was also prone to fungi *C. albicans* with inhibition zones (34–31 mm) and MIC value (30–40 µL/mL). Overall, the EOs of basil displayed a broad antimicrobial spectrum and exerted a much stronger antimicrobial effect against Gram-positive than against Gram-negative bacteria. These results are in accordance with the literature data [41,46]. The antimicrobial activity of the basil EO obtained by SFME is relatively higher than that obtained with HD. The EO of *O. basilicum* has been reported previously to have a good antimicrobial activity against a wide range of microorganisms [28,37,46,58,59]. Many authors have linked basil antimicrobial effects to the presence of high content of linalool. Thereby, Hussain *et al.* [33] reported that *O. basilicum* EO and linalool showed greater activity against bacterial strains than antifungal strains. Additionally, Hanif *et al.* [5] showed that Omani basil EO founded with higher linalool exhibited strong antibacterial activity against all the bacteria tested except *P. putida* and *P. aeruginosa*. Ouibrahim *et al.* [60] studied the antibacterial activity of Algerian basil EO, which is rich in linalool, against a wide range of Gram-negative and Gram-positive bacteria. They found that this oil is efficient in all tested bacterial strains except *P. aeruginosa*. Sienkiewicz *et al.* [61] demonstrated that basil oils containing mainly methyl chavicol (86.4%) can be widely used to eliminate clinical strains of *E. coli* found in different clinical conditions. Moghaddam *et al.* [62] revealed that the EO predominantly contains methyl chavicol (87.6%), which has antibacterial activity against all of the tested bacteria. These findings contradict those obtained by Lopez *et al.* [63] who reported that methyl chavicol has no relevance in the antimicrobial effects of the tested basil EOs in the vapor phase. By comparing the results of other researchers, it is possible to notice that for EOs tested, no significant correlation between the antibacterial activity and the percentage of the major components has been found. The different antibacterial activity exhibited by the oils, can be explained by either the synergistic effect of the different components in the oil and/or by the presence of other components that may be active even in small concentrations [43,64,65]. The antibacterial activity of EO from *O. basilicum* may be due in part to the presence of high content of linalool and methyl chavicol as the main components. However, between the two extracts of sweet basil, SFME-EO was relatively more active than HD-EO, which might be attributed to the high contents of (E)-methyl cinnamate, methyl eugenol, eugenol, and other oxygenated compounds in SFME-EO.

**Table 4:** Result of antimicrobial activity of sweet basil EO obtained by SFME and HD.

Tested Microorganisms	Inhibition Diameter ^1^ (mm ± Standard Deviation ^2^)		Minimum Inhibitory Concentration (µL/mL)	
	SFME-EO	HD-EO	SFME-EO	HD-EO
**Gram-Positive Bacteria**				
*Staphylococcus aureus* (ATCC 6538)	38 ± 1.5	33 ± 2.5	18	25
*Bacillus subtilis* (ATCC 6633)	37 ± 2.9	34 ± 1.2	18	25
**Gram-Negative Bacteria**				
*Escherichia coli* (ATCC 25922)	26 ± 2.1	22 ± 2.0	25	30
*Pseudomonas aeruginosa* (ATCC 14028)	29 ± 2.3	20 ± 3.2	20	30
**Yeast**				
*Candida albicans* (ATCC 10231)	34 ± 2.3	31 ± 2.3	30	40

^1^ Diameter of inhibition zone (mm) including disc diameter of 6 mm; ^2^ Each value is the mean ± SD of three replications.

## 3. Materials and Methods

### 3.1. Plant Materials

Sweet basil broken leaves (9.4% ± 0.4% of humidity) were purchased from *Dios herbarium of organic agriculture product*, F26410-Chatillon in Dios-France. *Ocimum basilicum* L. were collected from Egypt in 2011 and n° 28587.

### 3.2. Extraction Methods

#### 3.2.1. Solvent-Free Microwave Apparatus and Procedure

SFME was performed in a Milestone “DryDist” microwave laboratory (Milestone, Bergamo, Italy). This is a multimode 2.45 MHz microwave reactor with a maximum delivered power of 1000 W. Temperature was monitored by use of an external infrared (IR) sensor. During experiments, time, temperature, pressure, and power can be controlled. The experiment was conducted at atmospheric pressure with 150 g of *O. basilicum* immersed in water (600 g) during 30 min at 600 W. The plant material was heated by using a fixed power density of 1.0 W·g^−1^ for 30 min. A cooling system outside the microwave cavity condensed the distillate continuously with a Clevenger-type apparatus. Condensed water was returned to the flask and heating was continued at 100 °C until no more essential oil was obtained. The essential oil was collected, dried over anhydrous sodium sulfate, and stored at 4 °C until used.

#### 3.2.2. Hydro-Distillation Apparatus and Procedure

In this method, 150 g of *O. basilicum* immersed in 6 L water were submitted to hydro-distillation with a Clevenger-type apparatus for 1 h (until no more essential oil was obtained). The essential oil was collected, dried with anhydrous sodium sulfate, and stored at 4 °C until used.

### 3.3. GC and GC-MS Identification

#### 3.3.1. Gas Chromatography by Flame Ionic Detector (FID)

GC analysis was carried out using an Agilent 6890N gas chromatograph, under the following operation conditions: vector gas, Helium; injector and detector temperatures, 250 °C; injected volume, 1.0 µL; split ratio 1/100; HP1 column (J and W Scientific, Folsom, CA, USA), polydimethylsiloxane (50 m × 0.20 mm i.d., film thickness 0.33 µm; constant flow 1 mL/min) and INNOWAX (polyethyleneglycol, 50 m × 0.20 mm i.d. × film thickness 0.4 µm; Interchim, Montluçon, France). Temperature program 45–250 °C at 2 °C/min and 250 °C for 60 min. Retention indices were determined with C5 to C24 alkane standards as reference. Relative amounts of individual components are based on peak areas obtained without FID response factor correction. Three replicates were performed for each sample. The average of these three values and the standard deviation were determined for each component identified.

#### 3.3.2. Gas Chromatography-Mass Spectrometry Analysis

GC-MS analysis was carried out using an Agilent 6890N coupled to an Agilent 5973 MS (Agilent, Massy, France). Samples were analyzed on a fused-silica capillary column HP-1 (polydimethylsiloxane, 50 m × 0.20 mm i.d. × film thickness 0.33 µm; Interchim) and INNOWAX (polyethyleneglycol, 50 m × 0.20 mm i.d. × film thickness 0.4 µm; Interchim). Operation conditions: carrier gas, helium constant flow 1 mL/min, injector temperature, 250 °C, split ratio, 1:100, temperature program, 45 °C to 250 °C or 230 °C, at 2 °C/min then held isothermal (20 min) at 250 °C (apolar column) or 230 °C (polar column), ion source temperature, 230 °C; transfer line temperature, 250 °C (apolar column) or 230 °C (polar column); ionization energy, 70 eV; electron ionization mass spectra were acquired over the mass range 35–400 amu.

#### 3.3.3. Identification of the Components

Identification of the components was based on computer matching against commercial libraries (Wiley, MassFinder 2.1 Library, and NIST98), laboratory mass spectra libraries built up from pure substances, and MS literature data [66,67,68,69] combined with comparison of GC retention indices (RI) on apolar and polar column. RIs were calculated with the help of a series of linear alkanes C8–C24 on apolar and polar column (HP-1 and HP-INNOWAX). Compounds available in the laboratory were confirmed by external standard compound co-injection.

### 3.4. Physical Constants and Organoleptic Properties

Basil essential oils have been analyzed according to the standard method AFNOR [70]. The usual physical constants (specific gravity and refractive index) defining the EO have been determined at 20 °C. The organoleptic properties of the two basil EOs (SFME-EO and HD-EO) were analyzed by sensory evaluation. These tests were conducted by a panel comprising 10 graduate students from the University of Oran 1 Ahmed Benbella, Algeria. The samples presented to each panelist were evaluated as seen on Table 1 according to the following attributes: color, odor, and aspect.

### 3.5. Antioxidant Activity

The antioxidant activity of the *O. basilicum* EO was evaluated by the DPPH assay. DPPH (2,2-diphenyl-1-picrylhydrazyl) is a stable, highly-colored free radical that can abstract labile hydrogen atoms from phenolic antioxidants with concomitant formation of a yellow hydrazine (DPPH-H) [40]. The free radical-scavenging activity (RSA) of an extract can be expressed as the percentage of DPPH reduced by a given amount of extract. RSA was measured, following the modified method of Achat *et al.* [55]. Briefly, *O. basilicum* oil was subjected to serial dilutions in methanol to obtain different concentrations (2–20 mg/mL). 50 µL of each solution were added to 2 mL of a DPPH solution (2× 10^−4^ mol/L in methanol) and the mixture was left in the dark at room temperature for 30 min. The absorbance was then measured at 517 nm using a spectrophotometer. Free radical inhibition by DPPH was calculated in percent (%) according to the following equation:

$$\%\;\text{RSA}\;=\left(\frac{A_{0}-A_{i}}{A_{0}}\right)\times 100$$

A_0_: absorbance of DPPH solution without any antioxidant. A_i_: absorbance of DPPH solution after reaction with EO.

All experiments were performed in triplicate.

The IC_50_ value, which represents the EO concentration that caused neutralization of 50% DPPH radicals, was determined from the plot of the inhibition percentage against concentration. The capacity of each antioxidant was compared with the α-tocopherol (vitamin E) standard using the same methodology for various concentrations (0.2–5 mg/mL).

### 3.6. Heating Conditions

The effect of heating conditions was studied through the determination of the amount of total polar materials (TPMs) which assemble the different groups of altered compounds (free fatty acids, mono and diglycerides) and the oxidation and polymeric derivatives, all formed at temperatures below 180 °C (French law No. 86-857) [71]. 200 μg of either SFME-EO or HD-EO were added to 200 g of virgin olive oil (OO). The samples were then heated under domestic frying conditions, *i.e.*, at (180 ± 5) °C during several hours [56]. The temperature was monitored by a thermocouple (ATC-300) inserted directly into the domestic deep-fat electric fryers. All samples were analyzed before the first heating session and then every hour until degradation of the oil. The end of the heating assay was reached according to the maximum TPM value of 25% as tolerated by the French law (Article 3-3 of decree No. 86-857 of 18/07/86) and as mandatory in several countries [71]. The TPM percentage is determined using a Testo probe which tests quickly the quality of oil via the measurement of the dielectric constant. All the measurements were performed in duplicate.

### 3.7. Antimicrobial Activity

#### 3.7.1. Disc Diffusion Method

Antimicrobial activities of the essential oils were determined by the paper disc diffusion method [72,73]. For these assays, cultures of the following microorganisms were used: two Gram-positive (*Staphylococcus aureus* ATCC 6538 and *Bacillus subtilis* ATCC 6633), two Gram-negative (*Escherichia coli* ATCC 25922 and *Pseudomonas aeruginosa* ATCC 14028) bacteria, and one yeast (*Candida albicans* ATCC 10231). All microorganisms were supplied by the Algerian Pasteur Institute. The cultures of microorganisms were maintained on nutrient agar (NA) medium. Briefly, suspensions of the tested microorganisms (10^7^–10^8^ colony-forming units (CFU)/mL) were spread on the solid Muller-Hinton medium plates. Filter paper discs, 6 mm diameter (Whatman No. 1), were individually impregnated with 10 µL EO then placed onto the surfaces of the inoculated plates. A positive control containing microbial culture without the EO and a negative control containing only the medium were performed as well. At the end of the incubation time (24 h at 37 °C for bacteria and 25 °C for yeast), positive antibacterial and antifungal activities were established by the presence of a measurable inhibition zone and recorded in its width (mm) which includes the disc diameter. Each test was performed in three replicates.

#### 3.7.2. Determination of the Minimum Inhibitory Concentration (MIC)

For the determination of the MIC, which represents the concentration that completely inhibits the growth of microorganisms, a micro dilution broth susceptibility assay was used as recommended by Chemat *et al.* [74]. Dilution series were made in a concentration range from 10 µL to 40 µL/mL of the EO in sterile test tubes containing nutrient broth (NB) medium. Using a vortex, each tube was vigorously stirred in order to perfectly disperse the EO in the culture medium. Then, 15 µL of the tested microorganism suspension (10^7^–10^8^ colony-forming units (CFU)/mL) were inoculated into a test tube and incubated for 24 h in an oven at 37 °C for bacteria and 25 °C for yeast. A positive control containing microbial suspension without the EO and a negative control containing only the medium were performed. The MIC value was assimilated to the smallest concentration of the samples that did not become turbid. Each test was performed three times.

## 4. Conclusions

The solvent-free microwave extraction (SFME) technique has been compared with the conventional hydro-distillation (HD) method, for extraction of EO from Egyptian basil (*O. basilicum* L.) leaves. SFME was found to be highly effective enabling a considerable reduction in extraction time (30 min for SFME against 60 min for HD), providing an EO with yields similar to those of conventional HD. GC-MS results indicated that there were no significant differences between the constituents of EO obtained by SFME and those obtained by conventional HD. The results reported on the chemical composition of basil oil reveal that linalool represents the main compound in basil oil. Both essential oils SFME-EO and HD-EO indicate strong antimicrobial and low antioxidant activities and also a notable TPM effect. However, SFME-EO showed a better antimicrobial activity than HD-EO against all tested microorganisms, a greater antioxidant activity in the DPPH^•^ test and displayed an improved stability to virgin OO. SFME can be considered as a green technology that offers significant advantages over traditional hydro-distillation: shorter extraction times with similar yields, substantial energy and solvent saving, environmentally friendly approach, and lower cost. Thus, this method appears to be a good alternative for the extraction of EO from sweet basil for their applications in the food, pharmaceutical, and cosmetic industries.
